# Genetics (not only) for dummies

**DOI:** 10.1590/1678-4685-GMB-2016-0341

**Published:** 2017-05-29

**Authors:** Gabriel Adelman Cipolla, Marcia Holsbach Beltrame

**Affiliations:** 1 Department of Genetics, Universidade Federal de Paraná, Curitiba, PR, Brazil; 2 Department of Genetics, University of Pennsylvania, Philadelphia, PA, U.S.A

Genetics is a fast-growing and exciting field that ultimately touches everyone's life. The book *Genetics for Dummies* provides, in easy-to-understand language, an overview of the field of genetics and its current applications. This book is meant to help not only undergraduate students enrolled in genetics courses, but also any and every person interested in understanding more about genetics, *e.g.*, people with known or suspected family cases of genetic diseases, or simply those who are curious or concerned about genetic issues brought out in the press or popular media. Moreover, the book may also be useful for school teachers, providing them with a unique tool for classroom use, delivered in good-humored and accessible language.

The book is divided in five broad sections, totalizing 24 chapters. The first section primarily covers the classical field, *i.e.* Mendelian genetics, while the second is devoted to molecular genetics. The two following sections address how genetics may be applied to health, history, society and economy, also addressing ethical issues that arise as the field of genetics grows. In the last section, the author summarizes 10 hot topics from three different subjects: defining events, hot issues and hard-to-believe stories in genetics. Although these sections follow a traditional teaching sequence, parts or chapters can be read independently according to the needs or interests of the reader, who may be redirected to previous sections for basic information. Even though the latest edition of this book has been available for over half a decade, the book still delivers its main message as an introductory guide to genetics. Nevertheless, a reader with more knowledge of the area may enjoy its interesting material on the history or on new developments in the field, particularly in the final section of the book. There is no doubt, however, that the book could benefit from a new edition, bringing to light the latest advances, applications and issues in the field. Polemic topics have recently been brought out in the media, such as the genetic testing for mutations associated with breast cancer and the preventive mastectomy. Other issues are stirring increasing popular interest, for example the possibility of having one's whole genome sequenced and one's ancestry determined for about US$ 100.

The book provides an unbiased guide to polemic issues, demystifying controversies around genetically modified organisms (GMOs) and cloning, and deconstructing the biological basis of racism. In addition, the book also introduces the reader to scientific and academic career environments, describing the activities that take place in a lab, as well as the field of work for geneticists.

Topics such as GMOs, cloning, gene therapy, genetic counseling and stem cell applications are mainly addressed in the book in a U.S. context, while they could be compared to and/or discussed with regard to different realities. One striking case of how the author often focuses on American examples can be found in Chapter 19, when the grapefruit, a fruit barely known in some other countries, is used to illustrate the portfolio of crops that have been genetically modified through radiation. Another example of an issue that could be dealt with in more global terms shows up in Chapter 14, when, in exploring the genetics of cancer, the author limits epidemiological data to the U.S. Considering that such examples might not necessarily speak to the knowledge and experiences of all readers, this US-centric approach may fail to engage people from other parts of the world.

Since this book is supposed to be a general guide to genetics, each topic is briefly addressed. Hence, a more interested reader may eventually find the need to read more on a specific topic, in which case he or she would not find citations throughout the text, but only a few references to main websites in flagged paragraphs. These websites generally contain information about genetic diseases, as well as on some of the basics in the field of genetics. Nonetheless, neither the book nor the websites provide exercises, which would be important before moving on to another topic.

A widespread conceptual flaw frequently incurred by non-scientific articles and replicated in this book is related to the use of the term genetic code. The definition itself is correct when the process of translation is explained; however, there are several passages in which the term is misused. For example, when epigenetics is defined (page 348), the author uses genetic code as an equivalent for DNA sequence.

All in all, this book serves its purpose well: it covers the entire field of genetics, bringing out the most relevant discoveries and polemic issues until the end of the past decade, in an accessible style and playful language. Furthermore, as a low budget book, it reaches out to students and the general public with ease. Thus, given its content, scope and potential audience, providing an updated edition and translating this book into several different languages would be very useful, making this singular tool more complete and accessible for worldwide dissemination of contemporary genetic science, its discoveries and its applications.

